# Influence of SGLT2 Inhibitor Treatment on Urine Antioxidant Status in Type 2 Diabetic Patients: A Pilot Study

**DOI:** 10.1155/2021/5593589

**Published:** 2021-07-14

**Authors:** Diana Nabrdalik-Leśniak, Katarzyna Nabrdalik, Katarzyna Sedlaczek, Patryk Główczyński, Hanna Kwiendacz, Tomasz Sawczyn, Weronika Hajzler, Karolina Drożdż, Mirela Hendel, Krzysztof Irlik, Paweł Stelmach, Piotr Adamczyk, Andrzej Paradysz, Sławomir Kasperczyk, Tomasz Stompór, Janusz Gumprecht

**Affiliations:** ^1^Department of Internal Medicine, Diabetology and Nephrology in Zabrze, Faculty of Medical Sciences in Zabrze (41-800), Medical University of Silesia, Katowice, Poland; ^2^Students' Scientific Association by the Department of Internal Medicine, Diabetology and Nephrology in Zabrze, Faculty of Medical Sciences in Zabrze (41-800), Medical University of Silesia, Katowice, Poland; ^3^Department of Physiology in Zabrze, Faculty of Medical Sciences in Zabrze (41-800), Medical University of Silesia, Katowice, Poland; ^4^Doctoral School, Department of Pediatric Hematology and Oncology in Zabrze, Faculty of Medical Sciences in Zabrze (41-800), Medical University of Silesia, Katowice, Poland; ^5^Department of Urology, Faculty of Medical Sciences in Zabrze (41-800), Medical University of Silesia, Katowice, Poland; ^6^Department of Pediatrics, Faculty of Medical Sciences in Katowice (40-752), Medical University of Silesia, Katowice, Poland; ^7^Department of Biochemistry, Faculty of Medical Sciences in Zabrze (41-800), Medical University of Silesia, Katowice, Poland; ^8^Department of Nephrology, Hypertension and Internal Medicine, University of Warmia and Mazury in Olsztyn, Olsztyn (10-957), Poland

## Abstract

Sodium-glucose cotransporter 2 inhibitors (SGLT2i) have been recognized as potent antioxidant agents. Since SGLT2i are nephroprotective drugs, we aimed to examine the urine antioxidant status in patients with type 2 diabetes mellitus (T2DM). One hundred and one subjects participated in this study, including 37 T2DM patients treated with SGLT2i, 31 T2DM patients not using SGLT2i, and 33 healthy individuals serving as a control group. Total antioxidant capacity (TAC), superoxide dismutase (SOD), manganese superoxide dismutase (MnSOD), free thiol groups (R-SH, sulfhydryl groups), and catalase (CAT) activity, as well as glucose concentration, were assessed in the urine of all participants. Urine SOD and MnSOD activity were significantly higher among T2DM patients treated with SGLT2i than T2DM patients without SGLT2i treatment (*p* = 0.009 and *p* = 0.003, respectively) and to the healthy controls (*p* = 0.002 and *p* = 0.001, respectively). TAC was significantly lower in patients with T2DM treated with SGLT2i when compared to those not treated and healthy subjects (*p* = 0.036 and *p* = 0.019, respectively). It could be hypothesized that the mechanism by which SGLT2i provides nephroprotective effects involves improvement of the SOD antioxidant activity. However, lower TAC might impose higher OS (oxidative stress), and elevation of SOD activity might be a compensatory mechanism.

## 1. Introduction

Oxidative stress (OS) is defined as an imbalance between antioxidants and oxidants in favor of the latter, which leads to disruption of redox signaling and causes molecular damage [1]. Reactive oxygen species (ROS) constitute metabolites produced during physiological and metabolic processes and are neutralized by the antioxidant system [2]. OS occurs as a consequence of insufficient neutralization of ROS. It is well known that OS is involved in the development of diabetic vascular complications [2]. OS assessment can be based on measurement of ROS concentration, activities of antioxidant enzymes, and concentration of nonenzymatic antioxidative compounds [1]. Sodium-glucose cotransporter 2 inhibitors (SGLT2i), a new class of antidiabetic drugs, are thought to possess antioxidant properties. However, data on this issue come mostly from animal studies [3–6]. Inhibition of sodium-glucose cotransporter 2 (SGLT2) in proximal renal tubules increases urinary glucose excretion and decreases glycemia [7]. It is already known that besides the hypoglycemic effect, SGLT2i also provides cardiovascular benefits and nephroprotective effects through multiple biochemical pathways [8–13]. It has recently been revealed in large, randomized, placebo-controlled studies with canagliflozin [8–10], dapagliflozin [11], and empagliflozin [12, 13] that these drugs, in addition to their already proven cardioprotective properties, also exert substantial nephroprotective effects. These effects seem to be independent of glucose-lowering efficacy, but the exact mechanism of this action remains to be clarified. One of the hypotheses of these protective cardiorenal effects may be the potential for OS reduction preventing the free-radical generation [3–5] and enhanced antioxidant defense by increased activity of protective enzymes [14]. Unfortunately, studies assessing this phenomenon in patients treated with SGLT2i are scarce.

Superoxide is the primary ROS that links hyperglycemia and pathways engaged in developing vascular complications of T2DM. In turn, superoxide dismutase (SOD) is important for each cell because it leads to superoxide scavenging, and its isoform, manganese superoxide dismutase (MnSOD) localized in mitochondria, is considered the first-line defense against ROS [15]. Other important protective mechanisms include catalase (CAT) [16] and free thiol groups (R-SH, sulfhydryl groups) [17]. Activities of the enzymes mentioned above and concentrations of nonenzymatic factors can be measured to assess the redox status and intensiveness of an OS. It is also possible to measure total antioxidant capacity (TAC), which delivers information related to overall radical removal ability [18]. To date, most of the studies that demonstrate OS reduction caused by SGLT2 inhibition have been conducted on animal models with the use of blood and tissues of the studied animals [3–6]. There have also been attempts to examine OS in urine, which is tempting since it is a noninvasive, inexpensive, and simple to conduct assessment [19–24]. In the presented study, we hypothesized that treatment with SGLT2i would impact urinary antioxidant enzymes and nonenzymatic biomolecules as indicators of the antioxidant barrier. Urine examination was chosen because of its efficiency, material collection simplicity, and the possibility of using this method in further studies on a larger group of patients.

## 2. Materials and Methods

### 2.1. Subjects

This was an observational study of patients treated in the Outpatient Diabetology Clinic in the University Hospital in Zabrze, Poland. The consecutive eligible patients who fulfilled the inclusion criteria were invited to participate in the study. There were three groups of study participants: patients with T2DM treated with SGLT2i (group no 2), diabetic controls (i.e., patients with T2DM not treated with SGLT2i) (group no 1), and healthy controls (group no 3). Consecutive patients coming for a routine visit in the outpatient diabetology clinic who fulfilled the inclusion/exclusion criteria based on the medical documentation and interview were assigned into the groups. Healthy controls were recruited from the medical staff of the Hospital where the Outpatient Diabetology Clinic is located. The information about the recruitment process could be found on leaflets located in the Outpatient Diabetology Clinic.

The inclusion criteria for the study groups were age ≥ 18 years, T2DM of at least of 12 months duration, and treatment with SGLT2i for at least one month. The inclusion criteria for the diabetic controls were age ≥ 18 years, T2DM of at least of 12 months duration, and treatment with any antidiabetic drug except SGLT2i.

The exclusion criteria for the study groups were eGFR (estimated glomerular filtration rate) < 60 ml/min/1.73 m^2^, clinical signs of urinary tract infection and any other ongoing acute illness since these conditions could directly affect urine OS. The inclusion criteria for the control group were as follows: age ≥ 18 years and participants who had recent (assessed within less than three months) serum creatinine concentration within the reference range (assessed as a routine screening method performed in medical staff). The exclusion criteria for the control group was as follows: any chronic or acute illness at the time of enrolment into the study.

The medical history, including data regarding diabetes duration, demographic characteristics, comorbidities, and concomitant medications, was obtained from the medical records. HbA_1c_ (glycated hemoglobin A_1c_) was assessed as a routine part of outpatient diabetes management. Body mass and height were measured by standard methods. BMI (body mass index) was calculated among all participants, and the first morning urine void was collected on the day of the visit to the Outpatient Diabetology Clinic. Healthy volunteers had body mass and height measured by standard methods and BMI calculated, and first morning urine void collected on the day of informed consent was signed (leaflets advertising the study contained instructions on how to collect the first-morning urine void). The study protocol was exempt from the necessity of obtaining the approval of the Ethics Committee by the Medical University of Silesia due to its observational nature (KNW/0022/KB/33/19). Informed consent was obtained from all participants before inclusion into the study.

### 2.2. Biochemical Analysis—Blood Samples

Venous blood samples were taken as a routine part of diabetes management for evaluation of plasma HbA_1c_ and serum creatinine concentration with subsequent eGFR calculation during the patient's visit to the outpatient diabetology clinic. HbA_1c_ was measured with the HLPC (high-performance liquid chromatography) method [25], creatinine was measured with Jaffe's colorimetric assay [26] (Cobas Integra 800, Roche Diagnostics®), and eGFR was calculated according to CKD-EPI formula [27].

### 2.3. Biochemical Analysis—Urine Samples

The urine samples were collected from the fresh urine, the first void of the day into 75 ml sterile containers. All urine specimens were stored frozen at −80°C until testing at the end of the collection period. All urine samples were analyzed for glucose, creatinine with subsequent urinary albumin-to-creatinine ratio (UACR) calculation as well as SOD, MnSOD, TAC, R-SH, and CAT.

#### 2.3.1. Urine Glucose Concentration

Urine glucose concentration was measured by the colorimetric enzymatic method (Alpha Diagnostics® test) [28]. The glucose oxidase enzyme catalyzes the oxidation of glucose to gluconic acid and hydrogen peroxide [29]. The hydrogen peroxide oxidizes an oxygen acceptor to give chromogenic oxidation products; the intensity of its color is proportional to the amount of glucose [29]. Glucose concentration was expressed as milligram per decilitre of urine (mg/dl).

#### 2.3.2. UACR Measurement

Urine albumin concentration was measured with the turbidimetric immunoassay [30] (Cobas c501,Roche Diagnostics®), and urine creatinine concentration was determined by the Jaffe colorimetric assay [26]. UACR (mg/g creatinine) that has been calculated as albumin concentration (mg/L) divided by creatinine concentration (g/L) according to guidelines [30, 31].

#### 2.3.3. Urine Total SOD and MnSOD Activity

Urine total SOD activity, which is constituted by activities of all SOD isozymes, that is, extracellular SOD and both intracellular isoforms CuZnSOD and MnSOD, was measured according to the Oyanagui method [32, 33]. Superoxide anion, with the participation of xanthine oxidase, reacts with hydroxylamine creating nitrate ions, which after connection with naftylenodiamin and sulfanilic acid, produces color; for MnSOD measurement, potassium cyanide was used to deactivate other enzymes, including CuZnSOD and extracellular SOD [32, 33]. Reading was calculated against a blank probe consisted of bidestilled water, measured at a wavelength of 560 nm with Victor X3 Perkin Elmer® reader (Waltham, Mass., USA). This method is completely specific for SOD. The enzyme activity was expressed as nitrite units (NU) per ml of urine (NU/ml). One NU means the ability to 50% reduction of nitrate ion production in the presence of SOD.

#### 2.3.4. Urine TAC

Urine TAC was measured according to the Erel method. First, ABTS (2,2′-azinobis(3-ethylbenzothiazoline-6-sulfonic acid)) is oxidized to ABTS·^+^ radical cation by hydrogen peroxide [34]. Spontaneous reduction of ABTS·^+^ and thus, decolorization of its green solution is then accelerated by antioxidants, with a certain rate, depending on their concentrations and antioxidant capacities [34]. Color change was measured as a change of absorbance at a wavelength of 650 nm on the Victor X3 Perkin Elmer® reader. The result was determined from the standard curve, and an assay was calibrated by Trolox. TAC was expressed as mmol/l urine.

#### 2.3.5. Urine Free Thiol Groups

Urine free thiol group (R-SH, sulfhydryl groups) concentration was measured according to the Koster modified half-automatic method using the Victor X3 Perkin Elmer® reader at a wavelength of 405 nm [35]. This method is based on the reduction of DTNB (5,5′-ditiobis(2-nitrobensoid) acid) by chemical compounds containing sulfhydryl groups, and as a result, the yellow 5-tio-2-nitrobensoid anion is produced [35]. R-SH concentration was calculated from the standard curve; the sample was calibrated with glutathione. R-SH concentration was expressed as *μ*mol/l urine.

#### 2.3.6. Urine CAT Activity

CAT activity was measured according to the peroxidase method with Purpald (4-amino-3-hydrazino-5-mercapto-1,2,4-triazole) as chromogen [36]. This method is based on the reaction of the enzyme with methanol in the presence of an optimal concentration of hydrogen peroxide [36]. Produced formaldehyde was measured with Victor X3 Perkin Elmer® reader at the wavelength of 560 nm. Calibration was performed with formaldehyde. CAT activity was expressed as IU/l urine.

### 2.4. Statistical Analysis

The descriptive statistics of continuous variables were expressed as median (min-max) and 95% confidence interval (CI). The Shapiro-Wilk test was used to verify the normality of data distribution. For normally distributed variables, the independent Student *t*-test was applied for comparisons between groups. The *χ*^2^ Pearson's test was used for comparison between groups for categorized variables. The homogeneity of variance was checked using Levene's test. When more than two subgroups were compared, analysis of variance (ANOVA) and posthoc verification with the least significant difference test (LSD) and Bonferroni correction was utilized. Pearson's correlation coefficient measured the strength of the association between two variables. *p* value <0.05 was considered significant. Statistical analysis was conducted using Statistica Statsoft® version 13.3.

## 3. Results

### 3.1. Population Characteristics

The study included 101 participants. The demographic and clinical characteristics of the study subjects are described in Table 1. There were 37 patients (group 2) with T2DM treated with SGLT2i for at least 1 month (empagliflozin, *n* = 19 and canagliflozin, *n* = 18) and a control group of 31 patients (group 1) with T2DM not treated with SGLT2i and 33 healthy individuals (group 3) . There were significantly fewer men in the control group of healthy individuals than the patients treated (*p* = 0.012) or not treated (*p* = 0.0055) with SGLT2i. There was also a significant difference in age between the studied groups of patients and the healthy control group (*p* = 0.0012 for comparison of T2DM patients treated with SGLT2i and healthy controls and *p* = 0.0001 in case of comparing T2DM not treated with SGLT2i to healthy controls). There was also a significant difference in diabetes duration between patients treated or not with SGLT2i (*p* = 0.032). The only comorbidity among patients with diabetes was hypertension, and patients treated and not treated with SGLT2i did not differ in terms of the prevalence of hypertension, as well as the number and class of blood pressure-lowering medications. Healthy volunteers were not using any drugs in the long-term.

ACEI: angiotensin-converting enzyme inhibitors; ARB: angiotensin II receptor blockers; BMI: body mass index; eGFR: estimated glomerular filtration rate; HbA_1c_: glycated hemoglobin A_1c_; T2DM: type 2 diabetes mellitus; SGLT2i: sodium-glucose cotransporter 2 inhibitor; UACR: urine albumin-to-creatinine ratio; n: number of patients; *p* value: level of significance; ^∗^*χ*^2^, ^∗∗^ANOVA, ^∗∗∗^student *t*-test. Continuous variables are presented as median (min-max; 95% CI).

### 3.2. Antioxidant Status

Table 2 displays the comparison of urine antioxidant status and urine glucose concentration between both T2DM patient groups and healthy controls. Presented differences are independent of patients' age, which was proved in the ANOVA analysis.

T2DM: type 2 diabetes mellitus; SGLT2i: sodium-glucose cotransporter 2 inhibitors; SOD: superoxide dismutase; MnSOD: manganese superoxide dismutase; TAC: total antioxidant capacity; R-SH: sulfhydryl groups; CAT: catalase; n: number of patients. Continuous variables are presented as median (min-max; 95% CI).

LSD tests' significance: ^a^*p* < 0.01 (between (1) and (2)), ^b^*p* < 0.001 (between (2) and (3)), ^c^*p* < 0.01 (between (1) and (2)), ^d^*p* < 0.001 (between (2) and (3)), ^e^*p* < 0.01 (between (1) and (2)), ^f^*p* < 0.05 (between (2) and (3)), ^g^*p* < 0.05 (between (1) and (3)), ^h^*p* < 0.01 (between (2) and (3)), ^i^*p* < 0.001 (between (1) and (2)), ^j^*p* < 0.001 (between (1) and (3)), and ^k^*p* < 0.001 (between (2) and (3)).

**Figure 1:**
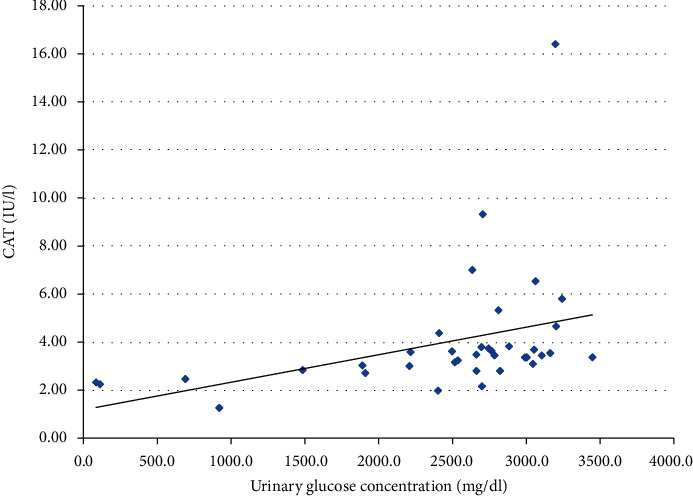
Correlation between urine CAT activity and urine glucose concentration in a group of patients treated with SGLT2i (*p* < 0.001).

**Figure 2:**
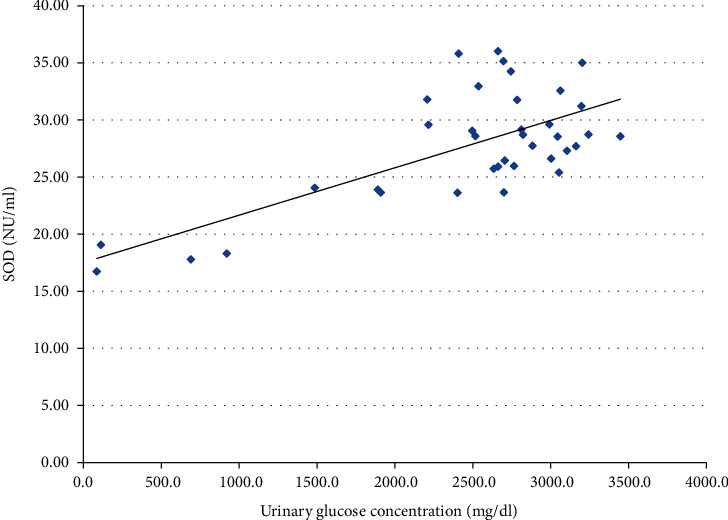
Correlation between urine SOD activity and urine glucose concentration in a group of patients treated with SGLT2i (*p* < 0.001).

**Figure 3:**
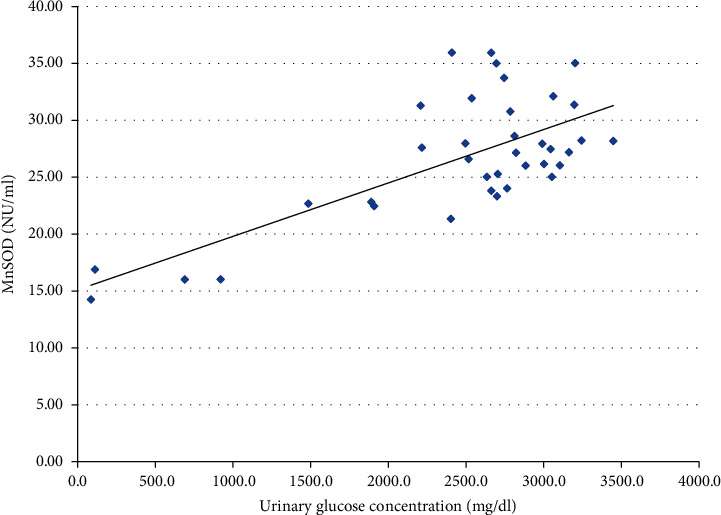
Correlation between urine MnSOD activity and urine glucose concentration in a group of patients treated with SGLT2i (*p* < 0.001).

We also performed the analysis of the possible relationship between glucosuria and the activities of the antioxidant enzymes. Analysis revealed significant positive correlations with CAT, SOD, and MnSOD (Figures 1, 2, and 3, respectively). SOD and MnSOD positive correlation stay in line with a significantly higher level of these enzymes in patients treated with SGLT2i.

## 4. Discussion

In this study, we report that the urine antioxidant status in patients treated with SGLT2i differs significantly from the one measured in T2DM patients not treated with SGLT2i, as well as from healthy controls. To date, only a limited number of studies have attempted to assess antioxidant enzyme activities in the urine of patients with T2DM, and neither one was performed in patients treated with SGLT2i. Hence, the direct comparison of our findings with other studies is not possible. Therefore, this discussion is focused on antioxidant status in blood and tissues in studies performed on animal models. In the presented study, among measured antioxidative enzymes, only CAT activity was comparable between the studied groups, whereas urine SOD and MnSOD activity was significantly higher among T2DM patients treated with SGLT2i compared to diabetic controls not receiving SGLT2i and to healthy controls. TAC was significantly lower in patients with T2DM treated with SGLT2i when compared to those untreated with SGLT2i and healthy subjects. In the case of R-SH, its concentration was significantly lower in patients with T2DM treated with SGLT2i than healthy controls. Yet, among patients with T2DM treated with SGLT2i and treated otherwise, the difference was not statistically significant.

On the other hand, R-SH concentration was significantly lower in patients with T2DM not using SGLT2i than healthy individuals. This observation might suggest that R-SH concentration is related rather to diabetes itself than to the mode of antidiabetic treatment. Yet, it needs further studies in larger groups of patients.

Since the results stemming from the animal studies implicate that SGLT2i mode of action is to reduce the OS [3–6], in this context, higher activity of the mentioned enzymes is surprising because this suggests that the OS is actually higher under this treatment. Previous studies performed in patients with diabetes revealed that TAC was lower among patients with chronic renal failure on maintenance dialysis [37], and total R-SH group concentration in urine of patients with diabetic kidney disease was similar to healthy subjects (although its serum concentration was lower in patients with diabetes) [20]. Similarly, other studies indicate that decreased systemic R-SH groups directly reflect increased whole body OS [38–41]. On the other hand, Shin et al., in their studies on animal models, demonstrated that dapagliflozin reduced OS by increasing MnSOD, Cu/ZnSOD, and catalase expression in renal tissues of animals with diabetic kidney disease [5]. As mentioned in the introduction, in addition to an already known protective effect in cardiovascular diseases, SGLT2i were recently found to have nephroprotective properties. ACEI (angiotensin-converting enzyme inhibitors), another class of nephroprotective agents, also contributes to protection against oxidative stress, for example, leading to the enhancement of SOD activity [42]. Therefore, one might hypothesize that for SGLT2i, the case might be similar. However, it must be kept in mind that this association was observed in blood, not in urine.

Also, the data on antioxidant enzyme activities in diabetes is controversial, and studies present equivocal results. Sundaram et al. [43] revealed in their experiment that plasma and erythrocyte SOD and CAT activities were decreased in patients with diabetes when compared to the nondiabetics. Similarly, Hartnett et al. [44] found reduced SOD activity in patients with diabetes. They suggested that increased activity of SOD might serve as a compensatory mechanism what was also mentioned as a reason for the elevation of plasma SOD activity in patients with T2DM in the study by Turk et al. [45].

While focusing on urine antioxidant activity studied to date in patients with diabetes, it is worth mentioning the study performed by Gul et al., who measured urinary antioxidant enzyme activities in T2DM subjects with urinary tract infection compared them with healthy subjects [46]. These authors revealed that both SOD and CAT activities were significantly lower in T2DM subjects with urinary tract infection than healthy controls, suggesting that elevated OS caused enzyme consumption [46]. Liu et al. demonstrated a higher concentration of OS biomarkers in the urine of T2DM patients with vascular complications as compared to those without it [24].

Additionally, since SGLT2i treatment leads to glucosuria, we performed a correlation analysis of glucosuria and antioxidant enzyme activity, which revealed significant positive correlations with respect to CAT, SOD, and MnSOD. These positive correlations are consistent with significantly higher SOD and MnSOD activity among patients treated with SGLT2i, whereas CAT activity seemed not to be affected by this treatment. Because no information regarding glucosuria and OS could be found in the literature, we can only hypothesize that glucosuria might stimulate OS and antioxidative defense by increasing SOD and MnSOD activity. Proximal tubules under treatment with SGLT2i are largely protected from glucose toxicity since their uptake by these cells is minimized. At the same time, tubular filtrate that reaches more distally located nephron segments is significantly enriched with glucose. Most of the recent interest concerning glucose handling by the kidney has been focused on the glucose uptake in the proximal nephron and the role of SGLT1 and SGLT2. Still, absorption of glucose in the loop of Henle and distal nephron segments (although much less important from a physiologic point of view) has also been confirmed [47, 48]. The apical glucose transporter (GLUT) transporters have been identified in the apical membranes of late nephron segment cells and were shown to be upregulated in the experimental models of diabetes; renal GLUT knockout can also induce glycosuria [49]. In the available literature, we did not find any data on glucose handling by the distal nephron following treatment with SGLT2i (although it has been shown that increased reabsorption of glucose by GLUT9 in proximal tubule following SGLT2i treatment is one of the mechanisms that competitively lowers uric acid reabsorption in this part of nephron) [50]. We hypothesize that increased glucose influx into the cells of distal nephron during the treatment with SGLT2i might inhibit Nrf2 (nuclear factor erythroid 2-related factor 2, the master transcription factor controlling defense against OS), thus enhancing OS in this part of nephron [51, 52]. It cannot be ruled out that to counterbalance this effect, certain antioxidant systems (possibly not controlled by Nrf2) might become enhanced in this part of nephron and result in increased activity of enzymes with an antioxidative potential in the final urine (as observed in our study). Several drugs which already used to treat diabetes or under the investigation as potential therapeutic agents, such as metformin, glucagon-like peptide-1 receptor (GLP1R) agonists, and bardoxolone, were shown to upregulate Nrf2 [53, 54]; we did not find such data for SGLT2i except for limited data on an animal model where empagliflozin is thought to promote the nuclear translocation of Nrf2 and limit the OS in the heart of mice with diabetes [3]. In our opinion, it is tempting to test such a hypothesis in an experimental study focusing on glucose handling by the distal nephron following the treatment with SGLT2i. Recently, redox balance has been characterized for the first time in saliva and blood of participants in different age groups revealing that antioxidant barrier decreases with age [55].Taking this information into account, an interesting direction of future research in this field would be inclusion of patients differing in age, especially elderly ones, since the human population is aging, and this favors diabetes complications occurrence.

In addition to OS, investigating the advanced end glycation products (AGEs) also seems a promising direction for future studies. AGEs are generated upon the nonenzymatic reactions between glucose and other sugars with proteins, lipids, or nucleic acids and participate in the pathogenesis of diabetic vascular complications [56–59]. AGEs can be related to oxidative stress because their formation starts under hyperglycaemic and/or oxidative stress conditions [60]. On the other hand, inhibiting the glycation process may be the way to limit the diabetes related complications [61]. Since there has been albumin glycation inhibition by metformin and glipizide demonstrated, in vitro study [62] maybe also SGLT2i may present this mode of action. The study performed most recently with another nephroprotective class of drugs—angiotensin II receptor blockers (ARB)—demonstrated their antiglycooxidant activity. These findings support the need for future studies in this area with the use of SGLT2i [63]. Given the fact that some literature reports suggest SGLT2i interact with AGEs during their formation, the next step in future studies could address this matter further [64–66]. For example, administration of empagliflozin for 4 weeks improved hyperglycemia and lowered HbA_1c_ level and resulted in a decreased expression of AGEs in kidneys of rats with streptozocin-induced diabetes [64]. Dapagliflozin ameliorated glucotoxicity in human renal proximal tubular epithelial cells by preventing AGE formation and synthesis of the proinflammatory cytokines such as transforming growth factor beta 1 (TGF-*β*1) and interleukin 8 (IL-8) [65]. A potential of AGEs to induce apoptosis of tubular epithelial cells through and interaction with AGE-receptor (RAGE) was ameliorated by the SGLT2 inhibition in a study conducted on cultured human renal proximal tubular epithelial cells [66]. Limitation of studies concerning the impact of SGLT2i on AGEs conducted on human cells suggests the need for further investigation in this field, with the inclusion of T2DM patients treated with SGLT2i.

## 5. The Limitations of the Study

The limitations of the study include differences in demographic characteristics of the examined groups (as there were more women among healthy individuals and those participants were younger than patients with T2DM) and differences in age and duration of T2DM between patients treated or not treated with SGLT2i. However, taking into account the mechanism of action of SGLT2i and its independence from endogenous insulin secretion, duration of diabetes should not affect the obtained results. Even though these limitations are acknowledged shortcomings of observational studies, it has been demonstrated recently that gender does not influence level of oxidative stress [55] but one must keep in mind that age differences could influence presented outcomes. It should also be noted that due to the small size of the group using ARBs, no additional analysis of their potential impact on the parameters of oxidative stress was performed. Given the fact that some recent literature reports suggest SGLT2i interact with AGEs during their formation and consequently affect the intensity of oxidative stress, the next step in future studies could be addressing this matter further.

## 6. Conclusions

In the presented study, we have demonstrated that treatment with SGLT2i influences urine antioxidant status in patients with type 2 diabetes. It could be hypothesized that the outcomes of the presented study indicate that the mechanism by which SGLT2i provides nephroprotective effects involves improvement of the SOD antioxidant defense. It would be of great value to elucidate the mechanism underlying the upregulation of SOD induced by SGLT2i in future studies. On the other hand, lower TAC might impose higher OS in the urine of patients treated with SGLT2i, and elevation of SOD activity might be a compensatory mechanism. Further studies are needed to replicate these results and resolve why TAC was lowered among patients treated with SGLT2i. It would also be interesting to assess oxidative urine status in prospective serial measurements in the same group of patients.

## Figures and Tables

**Table 1:** Demographic and clinical characteristics of the study subjects.

Variables	T2DM patients not treated with SGLT2is (1) *n* = 31	T2DM patients treated with SGLT2is (2) *n* = 37	Healthy controls (3) *n* = 33	*p* value
Male [*n* (% men)]	17 (55)	22 (59)	7 (21)	<0.01^∗^
Mean age [years]	60.0 (42.0-80.0; 95% CI: 56.7-62.7)	65.0 (39.0-78.0; 95% CI: 60.4-66.1)	51.0 (40.0-63.0; 95% CI: 49.3-53.2)	<0.001^∗∗^
Diabetes duration [years]	6.0 (0.00-20.00; 95% CI: 5.8-10.2)	13.0 (3.0 – 23.0; 95% CI: 27.8-31.1)	—	<0.01^∗∗∗^
HbA_1c_ [%]	7.9 (6.2-11.4; 95% CI; 7.6-8.6)	7.6 (6.4-10.0; 95% CI: 7.3-7.9)	—	>0.05^∗∗∗^
BMI [kg/m2]	29.7 (22.8-42.5; 95% CI: 28.5-31.6)	28.7 (21.9-52.7; 95% CI: 27.8-31.3)	26.6 (21.6-30.1; 95% CI: 25.4-27.0)	<0.001^∗∗^
eGFR [ml/min/1,73m^2^]	90.0 (62.3-106.1; 95% CI: 82.3-91.0)	92.5(62.1.3-113.7; 95% CI: 82.8.-92.6)	91,n(71.6-115.7; 95% CI: 85.8-93.7)	>0.05^∗∗^
Urinary albumin concentration [mg/l]	6.3 (0.2-16.5; 95% CI: 4.9-8.3)	4.2 (0.8-13.4; 95% CI: 3.8-6.2)	4.3 (1.2-9.4; 95% CI: 4.0-5.4)	>0.05^∗∗∗^
Urinary creatinine concentration [g/l]	0.5 (0.1-0.7; 95% CI: 0.4-0.5)	0.4 (0.1-0.7; 95% CI: 0.4-0.5)	0.4 (0.2-0.7; 95% CI: 0.4-0.5)	>0.05^∗∗∗^
UACR [mg/g]	13.3 (0.7-25.1; 95% CI: 10.6-15.3)	11.8 (1.8-21.8; 95% CI: 9.4-12.9)	10.4 (2.1-15.1; 95% CI: 9.3-11.4)	>0.05^∗∗∗^
ACEI or ARB [*n* (%)]	8 (25.8)	10 (27.0)	0 (0)	>0.05^∗^
Beta-blockers [*n* (%)]	4 (12.9)	5 (13.5)	0 (0)	>0.05^∗^
Hypertension [*n* (%)]	10 (32.0)	11 (30.0)	0 (0)	>0.05^∗^

**Table 2:** Comparison of antioxidant status and glucose concentration in urine among studied groups.

Variables	T2DM patients not treated with SGLT2i (1), *n* = 31	T2DM patients treated with SGLT2i (2), *n* = 37	Healthy controls (3), *n* = 33	*p* ANOVA
Total SOD [NU/ml]	25.2 (8.9-35.8; 95% CI: 21.1-26.2)^a^	28.5 (16.7-36.0; 95% CI: 26.1-29.4)^a,b^	23.2 (9.7-25.1; 95% CI: 20.2-25.2)^b^	<0.01
MnSOD [NU/ml]	22.6 (8.6-35.9; 95% CI: 19.1-24.4)^c^	27.1 (14.3-35.9; 95% CI: 24.9-28.5)^c,d^	21.6 (8.34-35.1; 95% CI: 18.8-23.9)^d^	<0.001
TAC [mmol/l]	9.9 (0.0-45.8; 95% CI: 7.9-14.6)^e^	7.9 (0.0-29.1; 95% CI: 6.5-11.7)^e,f^	9.2 (0.4-51.2; 95% CI: 8.4-15.2)^f^	<0.05
R-SH [*μ*mol/l]	1.7 (0.0-51.0; 95% CI: 4.5-16.2)^g^	1.9 (0.0-54.7; 95% CI: 3.4-11.8)^h^	13.6 (0.0-117.9; 95% CI: 10.8-30.6)^g,h^	<0.05
CAT [IU/l]	2.9 (1.0-8.4; 95% CI: 2.5-3.6)	3.4 (1.3-16.4; 95% CI: 3.2-4.9)	2.6 (0.6-9.5; 95% CI: 2.3-3.6)	>0.05
Urinary glucose concentration [mg/dl]	305 (49.0-3346.3; 95% CI: 269.9-897.7)^i,j^	2699.3 (85.7-3447.7; 95% CI: 2190.9-2742.0)^i,k^	2.8 (0.0-9.4 95% CI: 2.0-3.7) ^j,k^	<0.001

## Data Availability

The data presented in this study are available on request from the corresponding author. The data are not publicly available due to privacy.
